# Interpersonal motor resonance in autism spectrum disorder: evidence against a global “mirror system” deficit

**DOI:** 10.3389/fnhum.2013.00218

**Published:** 2013-05-23

**Authors:** Peter G. Enticott, Hayley A. Kennedy, Nicole J. Rinehart, John L. Bradshaw, Bruce J. Tonge, Zafiris J. Daskalakis, Paul B. Fitzgerald

**Affiliations:** ^1^Monash Alfred Psychiatry Research Centre, The Alfred and Central Clinical School, Monash UniversityMelbourne, VIC, Australia; ^2^Centre for Developmental Psychiatry and Psychology, School of Psychology and Psychiatry, Monash UniversityClayton, VIC, Australia; ^3^Centre for Addiction and Mental Health, University of TorontoToronto, ON, Canada

**Keywords:** mirror neurons, interaction, transcranial magnetic stimulation, primary motor cortex, electromyography

## Abstract

The mirror neuron hypothesis of autism is highly controversial, in part because there are conflicting reports as to whether putative indices of mirror system activity are actually deficient in autism spectrum disorder (ASD). Recent evidence suggests that a typical putative mirror system response may be seen in people with an ASD when there is a degree of social relevance to the visual stimuli used to elicit that response. Individuals with ASD (*n* = 32) and matched neurotypical controls (*n* = 32) completed a transcranial magnetic stimulation (TMS) experiment in which the left primary motor cortex (M1) was stimulated during the observation of static hands, individual (i.e., one person) hand actions, and interactive (i.e., two person) hand actions. Motor-evoked potentials (MEP) were recorded from the contralateral first dorsal interosseous, and used to generate an index of interpersonal motor resonance (IMR; a putative measure of mirror system activity) during action observation. There was no difference between ASD and NT groups in the level of IMR during the observation of these actions. These findings provide evidence against a global mirror system deficit in ASD, and this evidence appears to extend beyond stimuli that have social relevance. Attentional and visual processing influences may be important for understanding the apparent role of IMR in the pathophysiology of ASD.

## Introduction

The “mirror neuron hypothesis” is perhaps the most controversial recent theoretical account of autism spectrum disorder (ASD). Briefly, mirror neurons, which are cortical cells that fire during the performance and observation of behavior (Rizzolatti and Sinigaglia, 2010), were first identified in macaques (Di Pellegrino et al., 1992), and an analogous fronto-parietal “mirror system” has since been established in humans via a range of non-invasive neuroimaging and neurophysiological techniques (Rizzolatti and Sinigaglia, 2010). Beyond motor behavior, mirror systems have also been identified with respect to overlapping brain regions involved in the experience and observation of emotion, sensation, and pain (Keysers and Gazzola, 2009; Fitzgibbon et al., 2012). As mirror systems appear to simulate other's brain activity, they have been linked to a range of higher-order social cognitive processes, several of which are impaired in ASD. Accordingly, it has been suggested that dysfunction within mirror system circuitry, or of mirror neurons themselves, might contribute to ASD (Iacoboni and Dapretto, 2006; Oberman and Ramachandran, 2007; Rizzolatti and Fabbri-Destro, 2010). There are, however, arguments against such impairment (Gallese et al., 2011; Hamilton, 2013), and debate as to whether mirror systems are actually deficient in ASD.

Supporting evidence for mirror system dysfunction in ASD comes from a range of neurophysiological (Oberman et al., 2005; Bernier et al., 2007), neuroimaging (Dapretto et al., 2006; Hadjikhani et al., 2006, 2007), and brain stimulation studies (Theoret et al., 2005; Enticott et al., 2012c). These studies generally utilize an index of interpersonal motor resonance (IMR), which broadly refers to the activation of an individual's motor (or sensorimotor) system during the observation of another person's motor behavior (Uithol et al., 2011). Accordingly, IMR is typically considered a putative measure of mirror system activity. There seems to be little doubt that there are instances in which IMR is reduced in ASD, although increasingly there are studies that report no such deficit (Oberman et al., 2008; Raymaekers et al., 2009; Dinstein et al., 2010; Fan et al., 2010; Bastiaansen et al., 2011; Marsh and Hamilton, 2011). Among those studies that do report a deficit, perhaps most controversial is what these findings actually mean for our understanding of ASD; for example, whether they reflect an underlying neuropathophysiology that contributes to the clinical presentation, or are simply a neurobiological consequence of a lifetime of aberrant social engagement.

Given the proposed link between mirror systems and interpersonal understanding, there has been some interest in IMR during the observation of interactive or social behavior. For instance, there is evidence that IMR is enhanced during the observation of interactive behavior (Iacoboni et al., 2004; Oberman et al., 2007), particularly when there is a negative affective component (Enticott et al., 2011). Increased IMR is also seen during the observation of joint and complimentary actions (Newman-Norlund et al., 2007; Sebanz et al., 2007; Newman-Norlund et al., 2008). This is clearly of relevance to our understanding of ASD, which is characterized by difficulties in understanding other people and their interactions (Rapin, 1997). A study of 13 boys with ASD (and matched controls) showed that typical sensorimotor resonance [indexed via electroencephalogram (EEG) mu suppression] was evoked in ASD, but only when the intransitive hand gesture was performed by a familiar individual (e.g., parent) (Oberman et al., 2008). Although several factors may have underpinned this particular finding (e.g., familiarity, emotional relevance), this led the authors to speculate that the mirror system in ASD may be sensitive to the “social relevance” of the stimuli.

The current study used transcranial magnetic stimulation (TMS) to investigate IMR, a putative measure of mirror system activity, during the observation of individual and interactive hand movements among individuals with ASD. In line with the suggestion that social relevance may promote a typical mirror system response in ASD, it was hypothesized that IMR would be reduced in ASD for individual but not interactive conditions.

## Materials and methods

### Participants

Participants were 32 individuals with ASD [i.e., diagnosed with either autism (high-functioning) or Asperger's disorder] and 32 neurotypical (NT) controls (see Table 1 for participant demographics). All clinical participants had been diagnosed by an experienced clinician (psychologist, psychiatrist, or paediatrician) according to DSM-IV criteria (American Psychiatric Association, 1994). The diagnosis was confirmed via diagnostic report or through communication with the diagnosing clinician. Eleven of the clinical participants were medicated (6 selective serotonin reuptake inhibitor, 2 selective serotonin reuptake inhibitor /atypical antipsychotic, 2 selective serotonin reuptake inhibitor/atypical antipsychotic/benzodiazepine, 1 tetracyclic antidepressant, 1 atypical antipsychotic, 1 serotonin-norepinephrine reuptake inhibitor). Control participants all reported no history of neurological or psychiatric illness (including substance abuse). All participants met safety criteria for TMS and provided written informed consent. Ethical approval was granted by the human research ethics committees of Alfred Health, Monash University, and Southern Health.

**Table 1:** **Participant demographics**.

	**ASD**	**Controls**
n	32	32
Mean age in years (SD)	24.75 (8.11)	25.53 (6.36)
Gender (M:F)	24:8	23:9
Mean years of formal education (SD)^†^	14.67 (4.03)	17.48 (3.44)
Handedness (EHI) (R:L:A)	24:4:4	29:3:0
Mean KBIT-2 VIQ (SD)^*^	99.88 (17.72)	108.29 (13.54)
Mean KBIT-2 PIQ (SD)	107.78 (20.02)	112.52 (13.72)
Mean KBIT-2 FSIQ (SD)	104.63 (20.06)	112.13 (13.93)
Mean AQ (SD)^#^	30.97 (8.84)	13.29 (5.72)
Mean RAADS (SD)^#^	103.84 (39.29)	33.52 (22.86)
Mean DBC Total (SD)^*^	60.19 (21.64)	1.00 (−)
Mean DBC Autism Screen (SD)^*^	20.71 (7.16)	1.00 (−)

* p < 0.05,

† p < 0.01,

# p < 0.001.

EHI, Edinburgh handedness inventory (Oldfield, 1971); KBIT-2, Kaufman brief intelligence test, second edition; VIQ, verbal intelligence quotient; PIQ, performance intelligence quotient; FSIQ, full-scale intelligence quotient; AQ, autism spectrum quotient (Baron-Cohen et al., 2001); RAADS, Ritvo autism-aspergers diagnostic scale (Ritvo et al., 2008); DBC, Developmental Behaviour Checklist (Einfeld and Tonge, 2002).

### Materials

**Figure 1:**
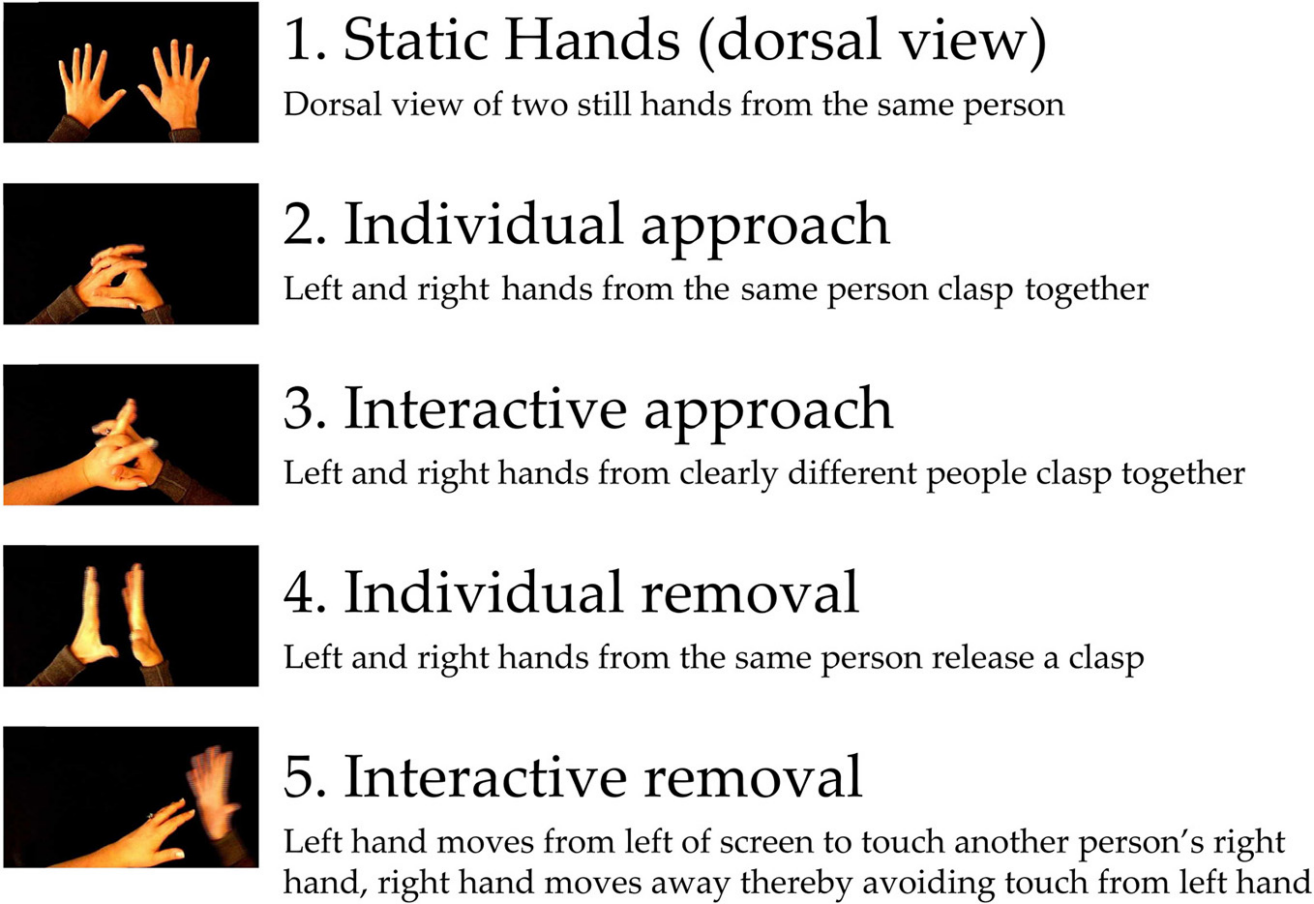
**Screenshots and descriptions of the five stimuli**.

Consistent with previous research (Gangitano et al., 2004; Fadiga et al., 2005; Theoret et al., 2005; Enticott et al., 2008a,b, 2012a,c), IMR was assessed by administering single pulse TMS to left primary motor cortex (M1), and recording responses in the right first dorsal interosseous via electromyography (EMG), during the observation of short videos featuring hand actions that involve the first dorsal interosseous. Stimuli were identical to those used in a previous study (Enticott et al., 2011), and involved five different videos of approximately 3–4 s duration (see Figure 1 for screenshots and descriptions): one demonstrating static hands (i.e., control/baseline condition), two demonstrating an individual's hand movements (involving the left and right hands from what was clearly the same person), and two demonstrating interactive hand movements (involving a left hand and a right hand from what were clearly two different people). The individual and interactive videos involved one in which the movement type was “approach” (i.e., right hand approaching the left hand) and one in which the movement type was “removal” (i.e., right hand moving away from the left hand). Ratings confirming the interactive and emotional content within each video are presented elsewhere, but essentially the “interactive removal” clip was rated as more emotional and more negative than the other clips (Enticott et al., 2011). The videos were designed such that the motor properties were matched for the two “approach” videos and for the two “removal” videos, thus enabling a valid comparison with respect to our index of mirror neuron activity. EMG equivalence within the two “approach” videos and the two “removal” videos was confirmed via separate EMG recordings, with <0.05 mV root mean square difference in EMG activity in the right first dorsal interosseous between the matched videos.

Single pulse TMS was administered using a Magstim-200 stimulator (Magstim Company Ltd., UK). EMG signals were amplified using PowerLab/4SP (AD Instruments, Colorado Springs, CO), and sampled via a CED Micro 1401 mk II analogue-to-digital converting unit (Cambridge Electronic Design, Cambridge, UK).

### Procedure

Participants were seated 120 cm in front of a 22″ widescreen (16:9) LCD monitor on which the video stimuli were presented (visual angle of video stimuli: 17.99 × 14.25°, although as seen in Figure 1 the hand actions comprised only a small proportion of the screen). Participants were not administered a formal test of visual acuity, but those that required eye glasses wore them throughout the procedure. Coil location for the stimulation of M1 was the scalp position that produced the largest amplitude MEP in the contralateral first dorsal interosseous while at rest. Resting motor threshold was the lowest stimulation intensity that produced motor-evoked potentials of at least 50 μV on 3/5 consecutive trials. Participants watched the video presentation, which was comprised of each of the five videos presented ten times in a quasi-random sequence. There was a 2000 ms interval between each clip, during which a black screen was displayed. For the static clip, a single TMS pulse was administered to left M1 approximately 2 s into the video. For the approach clips, a single TMS pulse was administered to left M1 immediately before the hands made contact. For the removal clips, a single TMS pulse was administered to left M1 immediately after the right hand started to move away from the left hand. This was based on optimal index finger/thumb aperture for generating sufficient IMR (Gangitano et al., 2001), and each involved index finger flexion/extension. TMS pulses during the video presentation were delivered at 120% of the RMT. Pulses administered during the video presentation were approximately 5–6 s apart. Triggering of the TMS stimulator was achieved via a light-sensor device that was placed over the upper left corner of the screen; embedded within each video was a brief white light that was hidden beneath the sensor and would appear at the designated frame, thus triggering the TMS pulse and EMG recording. Before and after the video presentation, participants were administered ten TMS pulses while at rest (4 s inter-stimulus interval) to determine whether the procedure itself, which might be considered a form of low-frequency repetitive TMS that could affect corticospinal excitability (Fitzgerald et al., 2006), induced any changes in corticospinal excitability.

### Data analysis

Trials in which there was evidence of tonic muscle activity within 200 ms prior to TMS administration were not included in the analyses (<0.5% of all trials). Consistent with our previous research (Enticott et al., 2011, 2012a,b,c), raw median MEP values were converted to reflect a percentage change relative to the baseline “static hands” condition [i.e., MEP percentage change (MEP-PC)], with a greater score indicative of greater IMR. Data screening of MEP-PC revealed non-normality, and we performed a square root data transformation. As a square root transformation cannot be performed for negative values (and some MEP-PC values were negative), prior to the transformation we added a constant of 100 to each of the values to ensure that they were all positive. Two extreme outliers (±3 *SD* from mean) following the transformation (interactive approach; both control participants) were adjusted to 0.01 above the next most extreme value. Normality was reassessed following transformation and found to be within acceptable limits. Data were analysed via a 2 (group: ASD vs. controls) × 2 (interpersonal type: individual vs. interactive) × 2 (movement type: approach vs. removal) mixed-model analysis of variance (ANOVA). Independent samples and paired samples *t*-tests were used for all follow-up analyses. We also used *t*-tests to examine whether there were any between-group differences in corticospinal excitability (i.e., raw median MEP values) across the various condition, and to determine whether the procedure itself had any influence on corticospinal excitability (i.e., raw median MEP values before and after the video presentation).

## Results

**Figure 2:**
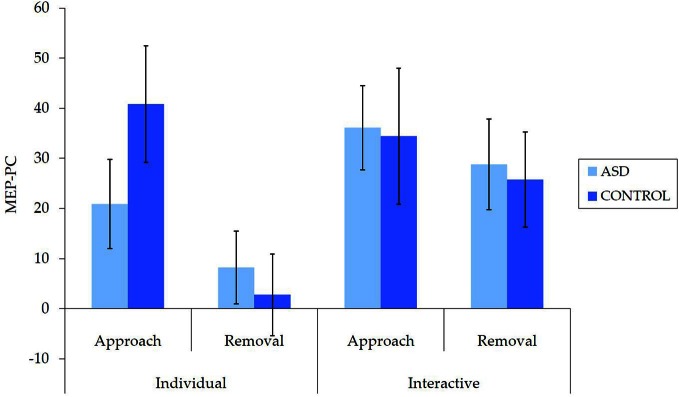
**Mean (±SE) (untransformed) MEP-PC by group for each condition; a greater score is indicative of enhanced IMR**.

Untransformed data are presented in Figure 2. For the overall mixed model analysis, there was no main effect of group, *F*_(1, 62)_ = 0.11, *p* = 0.915, η^2^_*p*_< 0.001, nor was there an interaction effect for movement type × group, *F*_(1, 62)_ = 0.70, *p* = 0.406, η^2^_*p*_ = 0.01 interpersonal type × group, *F*_(1, 62)_ = 3.13, *p* = 0.082, η^2^_*p*_ = 0.05, or interpersonal type × movement type × group, *F*_(1, 62)_ = 3.09, *p* = 0.084, η^2^_*p*_ = 0.05. Thus, concerning our hypothesis, there was no evidence for an *overall* IMR deficit in ASD for either the individual or interactive videos, nor was there evidence for any other reduction in IMR activity in ASD.

There were also a number of main and interaction effects that did not involve between-group effects. There was a main effect of interpersonal type, *F*_(1, 62)_ = 8.66, *p* = 0.005, η^2^_*p*_ = 0.12, with greater MEP-PC for interactive than individual movements, and a main effect of movement type, *F*_(1, 62)_ = 8.64, *p* = 0.005, η^2^_*p*_ = 0.12, with greater MEP-PC for approach than removal movement types. There was also an interaction effect between interpersonal type and movement type, *F*_(1, 62)_ = 4.82, *p* = 0.032, η^2^_*p*_ = 0.07. Subsequent analyses revealed greater MEP-PC for interactive removal than individual removal, *t*_(63)_ = −3.38, *p* = 0.001, *d* = 0.47, but no difference between individual approach and interactive approach, *t*_(63)_ = −0.54, *p* = 0.957, *d* = 0.01.

This pattern of results did not differ when including only right-handed participants: there was no main effect of group, *F*_(1, 51)_ = 0.13, *p* = 0.723, η^2^_*p*_ = 0.002, nor was there an interaction effect for movement type × group, *F*_(1, 51)_ = 1.62, *p* = 0.209, η^2^_*p*_ = 0.03, interpersonal type × group, *F*_(1, 51)_ = 3.96, *p* = 0.052, η^2^_*p*_ = 0.07, or interpersonal type × movement type × group, *F*_(1, 51)_ = 3.52, *p* = 0.066, η^2^_*p*_ = 0.07. This suggests that these findings are unlikely to have been affected by handedness, or between-group differences in handedness.

**Figure 3:**
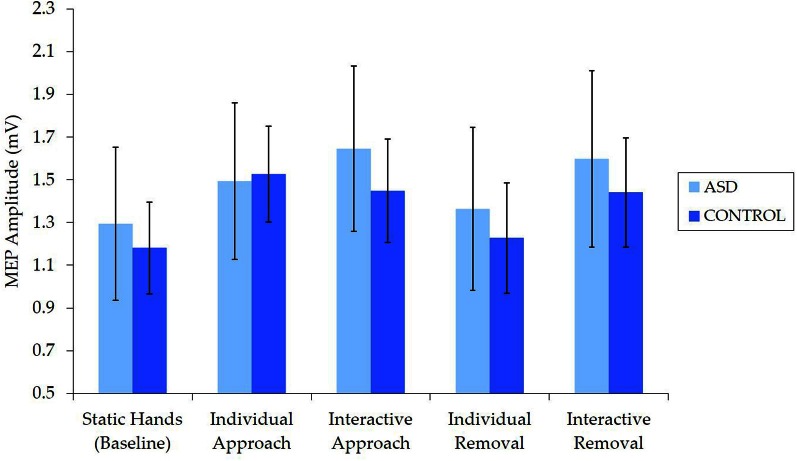
**Mean (±SE) raw (median) MEP by group for each condition, which demonstrates no differences in corticospinal excitability**.

Examination of raw MEP values indicated that corticospinal excitability was comparable for the ASD and NT groups (Figure 3). Independent samples *t*-tests revealed no between-group differences in median MEP amplitude for any of the five conditions [static hands: *t*_(62)_ = 0.27, *p* = 0.788, *d* = 0.07; individual approach: *t*_(62)_ = −0.77, *p* = 0.939, *d* = 0.02; interactive approach: *t*_(62)_ = 0.43, *p* = 0.667, *d* = 0.11; individual removal: *t*_(62)_ = 0.29, *p* = 0.769, *d* = 0.07; interactive removal: *t*_(62)_ = 0.32, *p* = 0.749, *d* = 0.08].

There was also no evidence to suggest that the TMS procedure altered corticospinal excitability in either group. Independent samples *t*-tests revealed no between-group differences in corticospinal excitability either before, *t*_(62)_ = 0.47, *p* = 0.640, *d* = 0.12 (ASD: *M* = 0.86 mV, *SE* = 0.11; NT: *M* = 0.79 mV, *SE* = 0.11), or after the video presentation, *t*_(62)_ = 0.58, *p* = 0.563, *d* = 0.15 (ASD: *M* = 0.85 mV, *SE* = 0.13; NT: *M* = 0.76, *SE* = 0.09). Similarly, paired samples *t*-tests revealed no differences in corticospinal excitability before and after the video presentation for either the ASD, *t*_(31)_ = 0.14, *p* = 0.889, *d* = 0.02, or NT group, *t*_(31)_ = 0.27, *p* = 0.792, *d* = 0.05.

As there were some between-group differences on demographic/cognitive variables (education, VIQ), we also performed Pearson correlations between measures of IMR and education/IQ. As presented in Table 2, these correlations were all weak and non-significant, suggesting that IMR was unlikely to have been influenced by these between-group differences.

**Table 2:** **Correlations between IMR and education/IQ (p-value in parentheses)**.

	**Individual approach**	**Interactive approach**	**Individual removal**	**Interactive removal**
Education	0.111 (0.384)	−0.004 (0.976)	−0.038 (0.766)	0.056 (0.662)
VIQ	0.040 (0.754)	−0.033 (0.798)	−0.178 (0.163)	0.016 (0.898)
PIQ	0.011 (0.935)	−0.034 (0.790)	−0.152 (0.233)	0.034 (0.794)
FSIQ	0.035 (0.786)	−0.033 (0.798)	−0.174 (0.173)	0.033 (0.795)

## Discussion

The current study utilized individual and interactive displays of hand movements to investigate IMR in ASD, which is broadly considered a means of testing the involvement of the “mirror system” in the neuropathophysiology of ASD. With respect to the observation of interactive hands, our hypothesis was supported: individuals with ASD did not show evidence of reduced IMR during the observation of interactive behavior. Contrary to our expectations, however, individuals with ASD did not exhibit evidence of reduced IMR compared to NT control participants during the observation of individual movements. Thus, in the current paradigm, there was no evidence for a reduction in IMR in ASD for either individual or interactive hand movements. Importantly, our analysis of raw MEP amplitudes confirmed that this was not attributable to baseline (or other) differences in the EMG response to TMS (i.e., corticospinal excitability), while the TMS procedure itself did not affect corticospinal excitability in either group. Additional findings that did not involve between-group effects were largely consistent with previous research (Enticott et al., 2011), and indicated greater IMR for interactive (relative to individual) and approach (relative to removal) videos, and an increase in activity for the interactive removal (relative to individual removal) video.

While there was no evidence to suggest a mirror system impairment in ASD, there were some interaction effects that approached significance; specifically, interpersonal type × group, and interpersonal type × movement type × group. These were each associated with a small to medium effect size, but raise the possibility of a type II error. Examination of mean data suggests the possibility of subtle differences in the pattern of responding for each group (e.g., enhanced interactive compared to individual in the ASD group). Taken together with other results, however, these again are not indicative of an “impairment” in ASD. Nevertheless, teasing out these subtle differences in mirror system activation will be an important consideration in future research.

These findings add to the controversy surrounding the role of mirror systems in ASD (Gallese et al., 2011; Hamilton, 2013) by further demonstrating that there are stimuli that evoke typical IMR in this population. Nevertheless, they are by no means entirely inconsistent with the literature, as there are a number of studies that report no mirror system impairments in ASD. For instance, Oberman et al. (2008) found that children with ASD showed appropriate sensorimotor resonance when observing grasping actions of a familiar person, while both Fan et al. (2010) and Raymaekers et al. (2009) found no evidence of reduced sensorimotor resonance among 20 children with ASD who observed hand movements. Several fMRI studies have also reported no abnormalities in the BOLD response in presumed mirror system regions among adults with ASD, with stimuli including transitive hand actions (Marsh and Hamilton, 2011) (*n* = 18 ASD), still images of hand gestures (Dinstein et al., 2010) (*n* = 13 ASD), and facial expressions (Bastiaansen et al., 2011) (*n* = 21 ASD). Studies that have and have not found these impairments in ASD seem to be comparable with respect to sample size, clinical characteristics, neuroscience techniques, and broad types of visual stimuli; thus, the heterogeneity of ASD might appear to be the most likely candidate to explain these inconsistent findings. The current results, however, cannot be attributed to such heterogeneity, as most of the participants in this study also completed a previous study in which IMR impairments in ASD were revealed during the observation of single hand transitive action (Enticott et al., 2012c). Interestingly, Theoret et al. (2005) found a deficit in IMR among individuals with ASD only when viewing a hand from an egocentric position, and it was suggested that this may reflect deficits in the representation of self. While the hands in the current study were positioned in this way, the use of multiple hands (including presentations involving hands from multiple people) may have reduced or eliminated any self-referential aspect to the stimuli.

These findings clearly argue against a global mirror system deficit in ASD, and thus these findings place substantive limitations on the “mirror neuron hypothesis of autism.” In the context of the previous literature, this study does not necessarily argue against any mirror system dysfunction in ASD. It does, however, suggest that there are situations in which IMR during action observation, a putative index of a mirror system response, is typical in ASD. It is now critical to establish the conditions under which IMR impairments are evident in ASD, and how this might relate to (or perhaps stem from) the behavioral phenotype of ASD.

There are other possible explanations regarding evidence for IMR deficits in ASD, and some of these would indeed argue against any level of mirror system dysfunction in ASD. For instance, it might be suggested that any observed deficits in IMR are not due to dysfunctional mirror system activity, but rather result from impairments in biological motion processing and attention in ASD that prevent subsequent mirror system activity. Concerning the former, there is evidence to suggest that individuals with ASD show atypical perception of biological motion, both at a behavioral level (e.g., reduced visual preference for biological motion; Klin et al., 2009; Annaz et al., 2012) and at a brain level (i.e., abnormal pattern of brain activation during biological motion perception; Kaiser and Pelphrey, 2012). Thus, it is conceivable that any deficit in IMR may actually result from earlier abnormalities in visual perception. This would not, however, provide an explanation for the current findings, where IMR during the observation of biological motion appeared largely typical, and certainly not significantly reduced.

The issue of attentional processing is difficult to disentangle from the perception of biological motion, but might provide a better alternative explanation for the current findings in the context of past literature. Clinically, individuals with ASD are generally thought to have a preference for objects over people (Rapin, 1997). Thus, when there is an object present (as in our previous study that showed IMR impairment; Enticott et al., 2012c), individuals with ASD may devote more attentional resources to the object and less to the human action (thus preventing IMR). This, however, fails to account for those studies demonstrating impairment in ASD when viewing intransitive actions (i.e., when there is no object present; e.g., Oberman et al., 2005; Theoret et al., 2005). Alternatively, and consistent with the weak central coherence account of ASD (which emphasizes enhanced local processing at the expense of global processing; Happe, 2005), they may attend to a specific feature of the object or the hand (e.g., the space between the fingers) rather than the active muscle region. In the current study, there were no objects present, perhaps encouraging individuals with ASD to entirely attend to the biological motion aspects (thereby promoting IMR). It may also be the case that the stimuli used in this study held greater interest or relevance for ASD participants than in other studies, meaning that they were more likely to sufficiently attend to the presentation (resulting in an IMR response that did not differ from controls). In some respects this is a motivational account, whereby participants with ASD need to be motivated to devote adequate attentional resources to the motion aspect of the stimuli. In any case, it would again argue against a specific mirror system deficit in ASD.

The issues of attention and processing of biological motion seem to be critical to truly understanding whether mirror systems play a role in the pathophysiology of ASD. At a minimum, future studies could integrate eye tracking techniques into existing neuroimaging or electrophysiological paradigms, or provide visual cues for ensuring that a particular aspect of biological motion is attended to. This issue is not specific to studies devoted to mirror circuitry, but would presumably apply to a range of neurobehavioral testing paradigms used commonly in ASD (e.g., tests of executive function or theory of mind). It is important to note that even if findings are modulated by these visual and attentional factors, it still does not necessarily argue against the mirror neuron hypothesis of autism, but would suggest an earlier and more general mechanism that leads to underactivity of the mirror system in ASD.

Limitations to this study include measurement of only the left cerebral hemisphere, a failure to probe individual participants about their interpretation of the stimuli, and the inclusion of medicated participants (although no between-group differences in corticospinal excitability were evident, medication effects cannot be ruled out). As noted, future research in this area should look to integrate neuroscience techniques (e.g., fMRI, TMS, EEG) with eye-tracking technology; this will go some way toward testing whether aberrant IMR is related to differences in visual attention (e.g., focusing on an object at the expense of a moving hand). A failure to detect group differences might also be due to the large variability of responses within each group, particularly for the *individual approach* condition. It is also important to note that the stimuli used here are very different to those used in classic “mirror neuron” studies among primates (which typically involve meaning, object-oriented actions). Thus, one might argue that the failure to find a difference is due to a failure to elicit mirror neuron activity in either group. While we cannot know whether true “mirror neurons” were indeed elicited by our stimuli, this is the case in all such non-invasive human research, and we have been careful to instead refer to IMR and mirror systems (i.e., increased motor cortical activity during the observation of motor behavior). It remains that both groups did demonstrate such increases in motor cortical activity. Nevertheless, the issue of whether these non-invasive paradigms are actually indexing (at least in part) true mirror neurons remains an important but elusive problem for this field of research.

In any event, these findings suggest that ASD is not characterized by a global deficit in mirror system activity, as there are conditions that produce largely appropriate levels of IMR in ASD. It remains to be determined why individuals with ASD do sometimes show reduced activity IMR during action observation, and whether this truly underpins the social and communicative deficits that characterize these conditions.

### Conflict of interest statement

The authors declare that the research was conducted in the absence of any commercial or financial relationships that could be construed as a potential conflict of interest.
